# L-Type Ca^2+^ Channels of NG2 Glia Determine Proliferation and NMDA Receptor-Dependent Plasticity

**DOI:** 10.3389/fcell.2021.759477

**Published:** 2021-10-21

**Authors:** Na Zhao, Wenhui Huang, Bogdan Cãtãlin, Anja Scheller, Frank Kirchhoff

**Affiliations:** ^1^Molecular Physiology, Center for Integrative Physiology and Molecular Medicine (CIPMM), University of Saarland, Homburg, Germany; ^2^Experimental Research Center for Normal and Pathological Aging, University of Medicine and Pharmacy of Craiova, Craiova, Romania

**Keywords:** L-type Ca^2+^ channels, oligodendrocyte lineage, myelination, neuron-NG2 glia synapses, neuronal plasticity, CACNA1C, CACNA1D

## Abstract

NG2 (nerve/glial antigen 2) glia are uniformly distributed in the gray and white matter of the central nervous system (CNS). They are the major proliferating cells in the brain and can differentiate into oligodendrocytes. NG2 glia do not only receive synaptic input from excitatory and inhibitory neurons, but also secrete growth factors and cytokines, modulating CNS homeostasis. They express several receptors and ion channels that play a role in rapidly responding upon synaptic signals and generating fast feedback, potentially regulating their own properties. Ca^2+^ influx *via* voltage-gated Ca^2+^ channels (VGCCs) induces an intracellular Ca^2+^ rise initiating a series of cellular activities. We confirmed that NG2 glia express L-type VGCCs in the white and gray matter during CNS development, particularly in the early postnatal stage. However, the function of L-type VGCCs in NG2 glia remains elusive. Therefore, we deleted L-type VGCC subtypes Cav1.2 and Cav1.3 genes conditionally in NG2 glia by crossbreeding NG2-CreERT2 knock-in mice to floxed Cav1.2 and flexed Cav1.3 transgenic mice. Our results showed that ablation of Cav1.2 and Cav1.3 strongly inhibited the proliferation of cortical NG2 glia, while differentiation in white and gray matter was not affected. As a consequence, no difference on myelination could be detected in various brain regions. In addition, we observed morphological alterations of the nodes of Ranvier induced by VGCC-deficient NG2 glia, i.e., shortened paired paranodes in the corpus callosum. Furthermore, deletion of Cav1.2 and Cav1.3 largely eliminated N-methyl-D-aspartate (NMDA)-dependent long-term depression (LTD) and potentiation in the hippocampus while the synaptic input to NG2 glia from axons remained unaltered. We conclude that L-type VGCCs of NG2 glia are essential for cell proliferation and proper structural organization of nodes of Ranvier, but not for differentiation and myelin compaction. In addition, L-type VGCCs of NG2 glia contribute to the regulation of long-term neuronal plasticity.

## Introduction

NG2 glia constitute about 5–8% of brain cells. They are uniformly distributed in the gray and white matter of the central nervous system (CNS) and act as oligodendrocyte precursor cells (OPC) (Nishiyama et al., 2009). In the adult CNS, NG2 glia are the major proliferating cell population outside neurogenic regions (e.g., subventricular zone and hippocampus). The cortical NG2 glia population is mostly maintained by local proliferation of existing NG2 glia (Hughes et al., 2013), whereas a small proportion of NG2 glia is generated from the subventricular zone migrating into the postnatal corpus callosum (Tripathi et al., 2011; Hill and Nishiyama, 2014). Within the NG2 population region-specific differences exist (Dawson et al., 2003). The proliferation rate of NG2 glia is faster in white than in gray matter with the shortest cell cycle in the corpus callosum (2.7 days), compared to spinal cord (4.4 days), and optic nerve (7.6 days), and almost 3 weeks (18.6 days) in the cortex (Zonouzi et al., 2011; Young et al., 2013).

NG2 glia express a variety of cell surface receptors and ion channels allowing them to detect and respond to a series of physiological activities, which regulate their own proliferation and differentiation (Larson et al., 2016). These ion channels and receptors, such as tetrodotoxin (TTX)-sensitive voltage-gated Na^+^ channels and glutamate receptors, were reported to have a dynamic expression level within their lifespan (Chittajallu et al., 2004; Zonouzi et al., 2011; Spitzer et al., 2019). NG2 glia can express voltage-gated Na^+^ channels in both white and gray matter of the postnatal brain. Blocking AMPA (α-Amino-3-hydroxy-5-methyl-4-isoxazolepropionic acid) receptors (AMPARs) or modifications of the GluA2 subunit of AMPARs in NG2 glia results in an impaired morphological development of OPCs and promoted proliferation and differentiation (Fannon et al., 2015; Chen et al., 2018).

Several voltage-gated Ca^2+^ channels (VGCCs, α1 subunits) are prominently expressed by NG2 glia: high-voltage activated L-type (Cav1.2 and Cav1.3), P/Q type (Cav2.1), and N-type (Cav2.2) as well as low-voltage activated T-type (Cav3.1 and Cav3.2) (Verkhratsky et al., 1990; Haberlandt et al., 2011; Zhang et al., 2014; Larson et al., 2016; Sun et al., 2016). Messenger RNA transcriptome and electrophysiology data revealed that L-type VGCC subtypes Cav1.2 and Cav1.3 predominate in early postnatal NG2 glia (Haberlandt et al., 2011), with decaying levels during the subsequent differentiation into mature oligodendrocytes (Fulton et al., 2010; Paez et al., 2010). Cav1.2 is required for multiple aspects of NG2 glia development throughout life of rodents. Its deletion during early postnatal stages restrains proliferation and maturation of OPCs, thus limiting myelination in the postnatal mouse brain (Cheli et al., 2016). Cav1.2 has been also described as a regulator for OPC maturation during the remyelination of the adult brain (Santiago González et al., 2017). Moreover, Cav1.2 deletion causes cell death in adult corpus callosum within a week after recombination. The remaining OPCs proliferate rapidly and fill the gap (Pitman et al., 2020). In addition, the L-type VGCC subtype Cav1.3 has been detected in NG2 glia by RNA sequencing as well (Zhang et al., 2014). Here, we aim to investigate the functional roles of L-type VGCC subtypes Cav1.2 and Cav1.3 by taking advantage of tamoxifen inducible NG2-CreERT2 knock-in mice to delete Cav1.2 and Cav1.3 selectively in NG2 glia.

## Materials and Methods

### Transgenic Mice

All experiments were carried out at the University of Saarland in strict accordance with the recommendations of European and German guidelines for the welfare of experimental animals. Animal experiments were approved by the Saarland state “Landesamt für Gesundheit und Verbraucherschutz” in Saarbrücken/Germany [animal license numbers: 72/2010, 65/2013, 34/2016 and FKI_cervical dislocation (§ 4)].

All mouse lines were maintained in C57BL/6N background and on a 12 hour (h) light/dark cycle at 20°C. Transgenic mice were housed at the animal facility of the CIPMM and fed a breeding diet (V1125, Sniff) *ad libitum*. NG2-EYFP knock-in mice (Karram et al., 2008), cacna1c floxed mice (Moosmang et al., 2003, 2005) and cacna1d-EGFP flexed mice (Satheesh et al., 2012) were kindly provided by Jacqueline Trotter (Mainz), Sven Moosmang (Munich) and Dusan Bartsch (Mannheim), respectively. The NG2 (cspg4)-CreERT2 knock-in mouse line had been generated previously (Huang et al., 2014). CAG-EGFP reporter mice (CMV-β actin promoter and loxP flanked CAT gene18 upstream of the EGFP cassette; Nakamura et al., 2006) were obtained from Leda Dimou (Ulm). The Rosa 26-td Tomato mouse line (Ai14) was obtained from Hongkui Zeng (Allen Institute) (Madisen et al., 2010). Mice were always heterozygous or wildtype at the NG2/cspg4 and reporter loci.

### Tamoxifen Administration and 5-Bromo-2-Deoxyuridine Treatment

Tamoxifen (T5648, Sigma-Aldrich, St. Louis, MO, United States) was dissolved in corn oil (Sigma-Aldrich) at the final concentration of 10 mg/mL. Mice were intraperitoneally injected with tamoxifen 7 days postnatally (P7) and P8 with a dosage of 100 mg/kg, once per day. In all experiments, both male and female mice were used. 5-Bromo-2-Deoxyuridine (BrdU) (Sigma-Aldrich, Taufkirchen bei München, Germany) (1 mg/mL) was dissolved in autoclaved water. To label proliferative cells, all tamoxifen treated mice received BrdU in drinking water for 3, 7, 10 and 14 days in the beginning of the 10th postnatal week and then were analyzed within 24 h after BrdU treatment was stopped (Rivers et al., 2008). Fresh BrdU water was given every two days.

### Tissue Preparation and Immunohistochemical Analysis

Mice were anesthetized by intraperitoneal injection of a mixed solution of ketamine (250 mg/kg bodyweight, Ketavet, Pfizer, Germany) and xylazine (50 mg/kg bodyweight, Rumpon, Bayer Healthcare, Germany) and intracardially perfused by 4% paraformaldehyde in 0.1 M phosphate buffer (pH 7.4) (Cãtãlin et al., 2017). After perfusion, brains, cervical spinal cords, and optic nerves were dissected and then post-fixed in the same fixative for 12–16 h at 4°C. Brains were immersed with PBS and 40 μm free-floating brain slices were cut with a vibratome (Leica VT1000S). Coronal forebrain sections at the hippocampal level (Bregma −1.34 to −1.82 mm), medial prefrontal cortex (PFC) (Bregma 2.22–1.54 mm) and anterior corpus callosum (Bregma 1.10–0.50 mm) as well as sagittal sections of the cervical spinal cords were collected.

Tissue slices were treated with blocking solution (0.3% Triton X-100 and 5% horse serum in PBS) for 1 h at room temperature (RT), and then transferred to primary antibodies to incubate for at least 12 h at 4°C. Brain and spinal cord slices were rinsed three times with PBS and then incubated in fluorescent secondary antibodies for 1 h at RT. DAPI (at the final concentration of 0.025 μg/mL) was added to secondary antibodies to detect nuclei. Primary and secondary antibodies were diluted in blocking buffer for use. Optic nerve tissue was treated as whole mount with modified blocking buffer including 1% Triton X-100.

For BrdU staining, brain slices were treated as previously described (Guo et al., 2010). Briefly, after finishing all the immunostaining steps except BrdU, brain slices were fixed in 2% paraformaldehyde for 30 min at RT and followed by denaturing in 2 M HCl at 37°C for 45 min. Then slices were rinsed with PBS and treated with anti-BrdU antibody as well as the secondary antibodies in blocking solution sequentially as described above.

The following primary antibodies were used: rabbit anti-GFP (1:1,000, Clontech, Mountain View, CA, United States), rat anti-PDGFRα (1:500, BD Pharmingen, San Diego, CA, United States), goat anti-PDGFRα (1:500, R&D Systems), mouse anti-CC1 (1:50, Calbiochem, Darmstadt, Germany), goat anti-Sox10 (1:50, R&D Systems), rat anti-BrdU (1:1,500, Abcam, Cambridge, United Kingdom), rabbit anti-cleaved caspase3 (1:500, Cell Signaling Technology, Danvers, MA, United States), rabbit anti-Caspr (1:1,000, Abcam), mouse anti-MBP (1:1,000, Biolegend, San Diego, CA, United States).

Donkey anti-mouse, goat, rabbit or rat secondary antibodies (1:1000) conjugated with Alexa488, Alexa555, Alexa633 or Alexa647 were purchased from Invitrogen (Grand Island, NY, United States). Goat anti-rabbit or rat secondary antibodies (1:500, conjugated with Cy5 were purchased from Dianova (Hamburg, Germany).

### Imaging Acquisition and Analysis

Confocal images were taken using a laser-scanning microscope (LSM-710, Zeiss, Oberkochen, Germany) with appropriate excitation and emission filters. Z-stack images were taken at 0.5–2 μm intervals and processed with ZEN software (Zeiss, Oberkochen, Germany). Cell differentiation and proliferation capacity was analyzed in 3–4 slices and for each animal both optic nerves were examined.

To capture overviews of brain sections, epifluorescent images were taken by a fully automated slide scanner (AxioScanZ.1, Zeiss, Oberkochen, Germany) equipped with an HBO lamp (HXP 120V, LEJ, Jena, Germany), appropriate excitation and emission filter sets, a Plan-Apochromat 10×/0.45 objective for pre-focusing and a Plan-Apochromat 20×/0.8 objective for fine focus image acquisition. The filter settings of excitation/emission wavelengths (in nm) were set as follows: 353/465 (DAPI), 488/509 (green), 548/561 (red) and 650/673 (infrared). Offline image stitching (7 μm stacks, variance projection) and further analysis were performed with ZEN software (Blue Edition, Zeiss). Cell counting was performed from 2–4 sections of overview images per mouse.

### Morphological Analysis

Confocal images were taken for morphological analysis from cortical NG2 glia in layer II/III with 0.438 μm intervals. Isolated PDGFRα^+^ and reporter^+^ cells were used for analysis. To quantify morphological changes on mutant NG2 glia, two approaches were performed, Sholl analysis and a binary particle analysis. Quantitative radial distribution of glial arborization processes were automatically evaluated by adapting a method first used to investigate the morphology of neurites for neurons (Sholl, 1953). In this study we used the logarithm of the radius and compared it to the logarithm of the ratio between the number of intersections (N) and the area of the corresponding circle (πR^2^), the formula is (log(R) vs. log(N/πR^2^) calculated by the Sholl analysis plugin of Fiji software. This method could well discriminate various cell types and is able to detect morphological differences of the same cell type in different regions (Milosevic and Ristanovic, 2007). The branching was analyzed by maximum intensity projections of 8-bit confocal images. The threshold of each individual cell was automatically adjusted before log-log analysis. The default minimum radius for the soma was set to 5 μm and every 1 μm increased one circle until the farthest point. The normalization was set to the area of each corresponding circle. In addition, we performed a binary particle analysis with the same raw data using the Particle Analysis function of Fiji and counted all particles of 0.2–4.15 μm^2^. In this analysis, the particles above 4.15 μm^2^ were excluded.

### *Ex vivo* Experiments

#### Acute Brain Slices Preparation

Mice were anesthetized with isofluran (Abbvie, Ludwigshafen, Germany) and after cervical dislocation the brain was removed and immersed in an ice-cold, oxygenated (5% CO_2_/95% O_2_, pH 7.4) slice preparation solution containing (in mM) 87 NaCl, 3 KCl, 25 NaHCO_3_, 1.25 NaH_2_PO_4_, 3 MgCl_2_, 0.5 CaCl_2_, 75 sucrose, and 25 glucose. Coronal vibratome slices of 300 μm thickness were prepared (Leica VT 1200S, Nussloch, Germany) and then transferred to a Nylon basket slice holder for incubation in artificial cerebral spinal fluid (ACSF) containing (in mM) 126 NaCl, 3 KCl, 25 NaHCO_3_, 15 glucose, 1.2 NaH_2_PO_4_, 1 CaCl_2_, and 2 MgCl_2_ at 32°C for 0.5 h. Subsequently, brain slices were taken out of the water bath and placed to RT with continuous oxygenation before use.

#### Electrophysiology

Slices were transferred to the recording chamber and continuously perfused with oxygenated ACSF containing (in mM)1 MgCl_2_ and 2.5 CaCl_2_ at a flow rate of 2–5 mL/min. NG2 glia were identified using a Zeiss microscope (Axioskop 2 FS mot, Zeiss) with a 40× water immersion objective and filter sets for YFP and GFP. Images were detected with a QuantEM 512SC camera (Photometrics, Tucson, United States) and displayed on a monitor. Whole-cell membrane currents were recorded by an EPC 10 USB amplifier (HEKA Elektronik GmbH, Lambrecht, Germany), low pass filtered at 3 kHz and data acquisition was controlled by Patchmaster software (HEKA). Patch pipettes (resistance, 7–9 MΩ) were fabricated from borosilicate capillaries (Outside diameter: 1.5 mm, inside diameter: 0.86 mm; Sutter Instrument Co., United States) using a Micropipette Puller (Model P-97, Sutter Instrument Co.). Patch pipettes were filled with an intracellular solution containing (in mM) 120 KCl, 2 MgCl_2_, 5 EGTA, 10 HEPES and 5 Na_2_ATP (pH∼7.2) for recording membrane currents of NG2 glia. The holding potential in voltage-clamp mode was −80 mV. Resting membrane potential was measured within 30 s after establishing the whole-cell recordings. To record L-type Ca^2+^ currents, pipette solutions contained (in mM): 120 CsCl, 1 MgCl_2_, 0.5 CaCl_2_, 5 EGTA, 10 HEPES, 5 Na_2_ATP and 20 tetraethylammonium chloride (TEA), its pH value was adjusted to ∼7.2 with CsOH. The extracellular solutions contained (in mM) 120 TEA, 10 HEPES, 5 CaCl_2_, 5 4-aminopyridine (4-AP) and 10 glucose. TTX (1 μM) was added in the bath to block voltage-gated Na^+^ channels. NG2 glia could be clearly distinguished from reporter^+^ pericytes due to their distinct morphologies (Ziskin et al., 2007). In NG2-CreERT2 mice, recombined NG2 glia differentiate into reporter^+^ oligodendrocytes. However, these oligodendrocytes could be easily distinguished in terms of their much lower membrane resistance and quite different morphology.

To record excitatory postsynaptic currents (EPSCs), NG2 glia in layer II/III of the somatosensory cortex were voltage-clamped while a concentric bipolar microelectrode (MicroProbes, United States) was placed in the layer V close to the border of layer IV, with a stimulus duration of 300 μs (Lalo et al., 2014). Patch pipettes were filled with a solution containing (in mM) 125 K gluconate, 20 KCl, 2 MgCl_2_, 0.5 EGTA, 5 HEPES and 5 Na_2_ATP, its pH value was adjusted to 7.2 with KOH. 6-cyano-7-nitroquinoxaline-2, 3-dione (CNQX, 30 μM) and D-(-)-2-amino-5-phosphonopentanoic acid (D-AP5, 30 μM) were applied *via* a custom-made perfusion system.

Compound action potentials (CAPs) were recorded in the corpus callosum as previously described (Crawford et al., 2009). Micropipettes filled with ACSF had a resistance of 1–3 MΩ. Inward responses were evoked in current-clamp mode by varying the intensity of stimulus pulses (0.2–4.0 mA) at 1 mm distance between recording and stimulation electrodes, with a stimulus duration of 200 μs. Sample sweeps were acquired every 5 s. Callosal conduction velocity was estimated by changing the distance from 2.5 to 0.5 mm between the stimulating and recording electrodes with a constant stimulus. To enhance the signal to noise ratio, we averaged the least 15 successive sweeps. Data analysis was performed with Igor pro 6.3.7.2 (WaveMetrics, Oregen, United States). The callosal axon conduction velocity was fitted with Graphpad Prism 7.0 (GraphPad Software, Inc., La Jolla, CA, United States).

Field excitatory postsynaptic potentials (fEPSP) were recorded in CA1 of hippocampus by stimulating Schaffer collaterals of CA3. Picrotoxin (50 μM) was perfused in the bath to inhibit ionotropic γ-aminobutyric acid type A receptors (GABA_*A*_Rs). Stimulus duration was 200 μs, current injection was 30–80 μA. Micropipettes filled with ACSF had a resistance of 1–3 MΩ. To elicit LTD, low frequency stimulation (LFS) was performed at 1 Hz for 15 min (Luscher and Malenka, 2012). To evoke LTP, triple θ-burst stimulation (TBS3) was used (Wang et al., 2016). TBS consisted of 10 bursts (4 pulses each burst, 100 Hz) delivered at an interburst interval of 200 ms, and repeated once at 10 s. The stimulation intensity was adjusted to evoke ∼30–60% of the maximum response. Waveform analysis was performed by Igor pro 6.3.7.2. The statistical analysis was conducted in Graphpad Prism. All experiments were conducted at RT.

### Ca^2+^ Imaging

In acutely isolated brain slices of transgenic mice, fluorescent reporter-positive NG2 glia were loaded with 100 μM Fluo-4 potassium salts *via* the patch pipette during whole-cell patch-clamp recordings. Fluo-4 potassium salts (ThermoFisher Scientific Inc.) were prepared into stock solution at a concentration of 2 mM in water and then stored at −20°C. Before every experiment, Fluo-4 was dissolved into pipette solution containing (in mM) 125 potassium gluconate, 20 KCl, 2 MgCl_2_, 0.05 EGTA, 10 HEPES and 5 Na_2_ATP and 0.018 CaCl_2_, its pH value was adjusted to 7.2 with KOH. The dye was allowed to diffuse into fine processes at least for 30 min before imaging. The holding potential in voltage-clamp mode was at −80 mV. Zeiss microscope equipped with 63× water immersion objective was used to visualize the regions of interest and the images were captured with a QuantEM 512SC camera. Imaging acquisition was controlled by Imaging Workbench software 5.2.20.6 (INDEC BioSystems, United States) at 20 Hz. To evoke signals, cells were depolarized from −110 to 10 mV for 100 ms by 25 pulses (Haberlandt et al., 2011). The baseline was recorded for 10 s before stimulating. Photobleaching was corrected by a mono-exponential curve. The equation: I(t) = A^∗^exp(-t/τ) where the intensity I(t) is a function of time t. The mono-exponential approach considers a homogeneous fluorochrome population with the rate of photobleaching and initial intensity A. Changes in [Ca^2+^]_*i*_, measured as changes in fluorescence intensity (ΔF), were calculated by ΔF/F_0_ = (F - F_0_)/F_0_. F_0_ is the baseline fluorescence. Data analysis was performed with Image J and custom-made scripts with Matlab R2014a (MathWorks, United States) and Graphpad Prism.

### Statistical Analysis

Statistic differences were analyzed using the unpaired two-tailed student *t*-test for two group comparison and one-way ANOVA for multi-group comparison. The levels of significance were set as ^∗^*P* < 0.05, ^∗∗^*P* < 0.01, ^∗∗∗^*P* < 0.001. Data are shown as mean ± SEM. Mann-Whitney tests were used when data did not show a Gaussian distribution.

## Results

### Postnatal Removal of L-Type Ca^2+^ Channel Subtypes Cav1.2 and Cav1.3 in NG2 Glia

**FIGURE 1:**
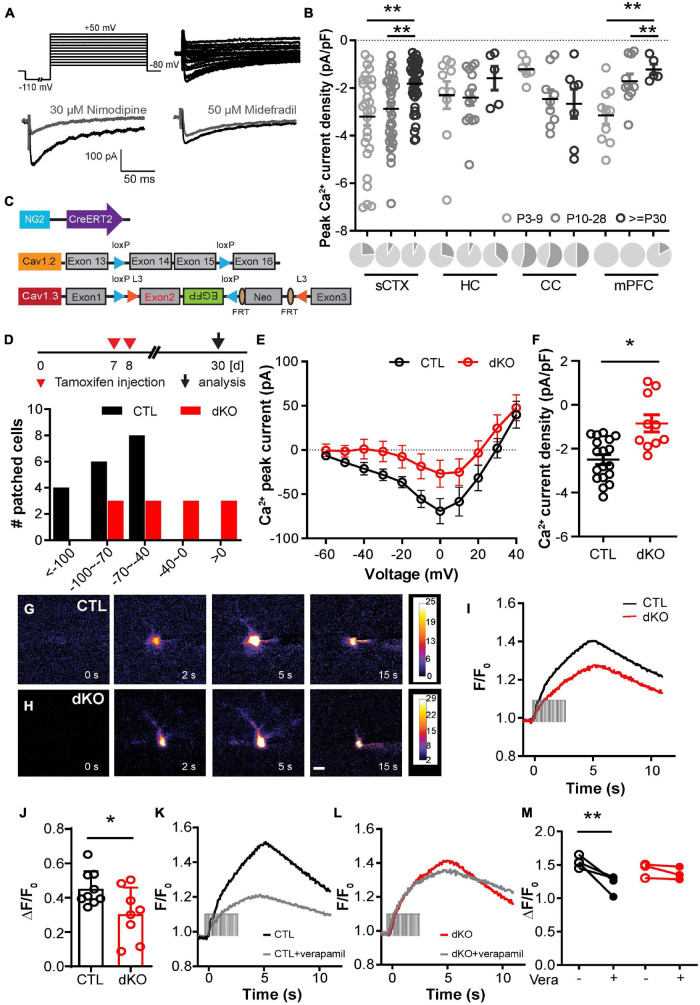
Conditional deletion of L-type voltage-gated Ca^2+^ channel subtypes Cav1.2 and Cav1.3 in NG2 glia. **(A)** Ca^2+^ current recordings. Peak inward currents of NG2 glia in the somatosensory cortex were strongly blocked by L-type Ca^2+^ channel blocker nimodipine and only weakly blocked by T-type blocker mibefradil. **(B)** L-type Ca^2+^ peak current densities in different brain regions (sCTX, somatosensory cortex; HC, hippocampus; CC, corpus callosum; mPFC, medial prefrontal cortex) during CNS maturation. Within the first month postnatally the expression levels of VGCC decreased by about 30% in cortical areas, but appeared largely unchanged in HC and CC. Each pie chart represents the proportion of NG2 glia with Cav expression (in light gray) and non-Cav expression (in dark gray). **(C)** Genetically modified mouse lines for NG2 glia-specific Cav1.2 und Cav1.3-specific gene deletion. **(D)** The upper panel depicts the tamoxifen injection protocol. Tamoxifen was administered at postnatal 7 and 8 days, the electrophysiological analysis was performed 3 weeks later. The bottom panel depicts the distribution of peak Ca^2+^ current amplitudes of control and double knockout (dKO) mice. None of the dKO NG2 glia had larger peak Ca^2+^ currents than −100 pA currents. The number of dKO NG2 glia with currents between −100 pA to −40 pA were reduced compared to controls. Half of the dKO NG2 glia displayed peak currents smaller than −40 pA. **(E)** I/V relationships of control and mutant mice including Cav1.2/Cav1.3 single and dKO mice. **(F)** The peak Ca^2+^ current density of mutant NG2 glia is strongly reduced in comparison to controls. Each datapoint represents recordings from a single NG2 glial cell. More than three mice per group were studied. **(G,H)** Ca^2+^ imaging of NG2 glia after loading with Fluo 4 potassium salts by whole-cell patch-clamp recording. Ca^2+^ elevations of NG2 glia in the somatosensory cortex were evoked by successive depolarization to 10 mV in control **(G)** and dKO mice **(H)**. The basal fluorescence prior to 0 s was subtracted from all the images shown here. Scale bar = 5 μm. **(I,J)** Somatic Ca^2+^ elevation upon depolarization was significantly decreased in mutant NG2 glia. **(K,L)** Pharmaceutical inhibition of Ca^2+^ rises by verapamil in control **(K)** and mutant NG2 glia **(L)**. **(M)** Verapamil could largely abolish the Ca^2+^ signals in control **(K)**, but it had no effects on mutant NG2 glia. *p < 0.05; **p < 0.01; ***p < 0.001.

To study the expression of L-type voltage-gated Ca^2+^ channels (VGCCs) in NG2 glia in different brain regions during CNS development, we analyzed reporter^+^ NG2 glia in the white matter (corpus callosum, CC) and gray matter (somatosensory cortex, sCTX; medial prefrontal cortex, mPFC, and hippocampus, HC) by whole-cell patch clamp recordings using acute brain slices of NG2-EYFP mice (Karram et al., 2008) and NG2-CreERT2-td Tomato (Huang et al., 2014) mice [from postnatal 3 days (P3) up to 12 weeks]. In order to record isolated Ca^2+^ currents, we increased [Ca^2+^] in the extracellular solution to 5 mM and blocked voltage-gated Na^+^ channels by tetrodotoxin (TTX) and K^+^ channels by Cs^+^, tetraethyl ammonium (TEA) and 4-aminopyridine (4-AP) (Haberlandt et al., 2011). Depolarizing voltage steps from −60 to +50 mV elicited respective Ca^2+^ currents. Inward currents could be evoked starting from −40 mV with a peak at about 0 mV and decline with increasing voltage steps (Figure 1A). In the somatosensory cortex, the peak Ca^2+^ inward currents were vastly blocked by L-type Ca^2+^ channel blocker nimodipine (87.6% ± 10.5%, *n* = 4 cells from 3 mice), while the T-type Ca^2+^ channel blocker mibefradil only partially reduced the peak inward currents by 14.3% ± 6.5% (*n* = 5 cells from 3 mice, Figure 1A). It indicated that L-type voltage-gated Ca^2+^ channels were predominantly expressed in NG2 glia. By examining NG2 glia in different brain regions during CNS development, we found that not all NG2 glia expressed L-type Ca^2+^ channels in the investigated brain regions (sCTX, HC, CC, and mPFC) and that the proportion varied between age and region (proportion of non-Cav expressing NG2 glia shown in dark gray, Figure 1B). It is worth noting that, the proportion of non-Cav expressing callosal NG2 glia is higher than in the cortical population. By analyzing Cav-expressing NG2 glia in various brain regions, we observed age-dependent changes of the peak Ca^2+^ current density. It decreased significantly with age in the cortex, while it large stayed constant in hippocampus and white matter. In the somatosensory cortex, the expression level of L-type VGCCs in NG2 glia reached its maximum in the first 2–3 postnatal weeks (P3–9, -3.20 ± 0.37 pA/pF, *n* = 28 cells; P10-28, −2.88 ± 0.26 pA/pF, *n* = 33 cells; >P29, −1.82 ± 0.15 pA/pF, *n* = 42 cells), and then decreased from the fifth postnatal week (Figure 1B). In the medial PFC, we observed a similar peak Ca^2+^ current density that decreased remarkably in the second week after birth (P3–9, −3.15 ± 0.37 pA/pF, *n* = 10 cells; P10–28, -1.71 ± 0.31 pA/pF, *n* = 10 cells; >P29, −1.22 ± 0.22 pA/pF, *n* = 5 cells). The membrane capacitance of NG2 glia in the cortex, hippocampus and corpus callosum did not vary during CNS development, with an average value of 20–30 pF (data not shown). These data demonstrate that L-type voltage-gated Ca^2+^ channels are widely expressed in most NG2 glia in all analyzed brain regions, including gray and white matter during CNS maturation. Specifically, the expression level of L-type VGCCs in the cerebral cortex significantly decreased adult mice.

To investigate the functional roles of Cav1.2 and Cav1.3 in NG2 glia, we took advantage of the tamoxifen-inducible Cre DNA recombinase to ablate Cav1.2 (*cacna1c*) and Cav1.3 (*cacna1d*) specifically in NG2 glia by crossbreeding homozygous floxed Cav1.2 (Moosmang et al., 2003, 2005) and flexed Cav1.3 (Satheesh et al., 2012) to NG2-CreERT2 knock-in mice (Huang et al., 2014; Figure 1C). These triple transgenic mice were crossbred to CAG-EGFP reporter mice (Nakamura et al., 2006) to label recombined cells. As our previous data showed peak expression of L-type VGCCs in NG2 glia in the first postnatal week (Figure 1B), we evoked recombination by intraperitoneal injection of tamoxifen once per day at postnatal days 7 and 8 (Figure 1D). In total, we recorded 12 mutant NG2 glial cells. In three of them we did not detect inward currents when depolarized to 0 mV (>0 pA). None of the cells showed inward currents larger than −100 pA currents. Most of the cells had inward currents between −100 pA to 0 pA. L-type Ca^2+^ currents of NG2 glia were reduced in all mutant mice (dKO) compared to controls at three weeks after tamoxifen administration (Figures 1D–F). Peak Ca^2+^ current density of controls (CTL) was −2.50 ± 0.22 pA/pF, and was reduced to −0.85 ± 0.39 pA/pF in dKO mice (Figure 1F). After loading Fluo 4 potassium-salt during whole-cell recordings, single-cell Ca^2+^ imaging showed that Ca^2+^ influx *via* the plasma membrane of NG2 glia induced a Ca^2+^ release, that could be largely inhibited by L-type VGCC blocker verapamil (ΔF/F_0_ CTL vs. verapamil, 0.54 ± 0.04 vs. 0.22 ± 0.07, *n* = 4 cells, Figure 1K). Thereby, substantiating that voltage-gated Ca^2+^ influx into NG2 glia occurs mainly through L-type VGCCs (Figures 1G,K). Upon depolarization Ca^2+^ elevation in mutant NG2 glia was smaller than in control (CTL 0.45 ± 0.03 vs. dKO 0.31 ± 0.05, control from 9 cells of 5 mice, dKO from 8 cells of 4 mice, Figures 1H–J). However, we still could observe Ca^2+^ induced Ca^2+^ release in mutant NG2 glia upon depolarization, while verapamil had no effect on Ca^2+^ elevation (ΔF/F_0_ dKO vs. verapamil, 0.43 ± 0.06 vs. 0.37 ± 0.06, *n* = 3 cells, in Figures 1L,M). Probably T-type Ca^2+^ channels compensate in the absence of L-type VGCCs. These data indicate that Ca^2+^ influx into NG2 glia mainly occurs *via* the activation of L-type voltage-gated Ca^2+^ channel. Removal of Cav1.2 and Cav1.3 from NG2 glia at early postnatal stages did not affect cellular activities. T-type voltage-gated Ca^2+^ channels might be upregulated in Cav1.2/1.3 deficient NG2 glia.

### Ablation of Cav1.2 and Cav1.3 Inhibited Proliferation of Cortical NG2 Glia, but Did Not Affect Their Differentiation Into Mature Oligodendrocytes

**FIGURE 2:**
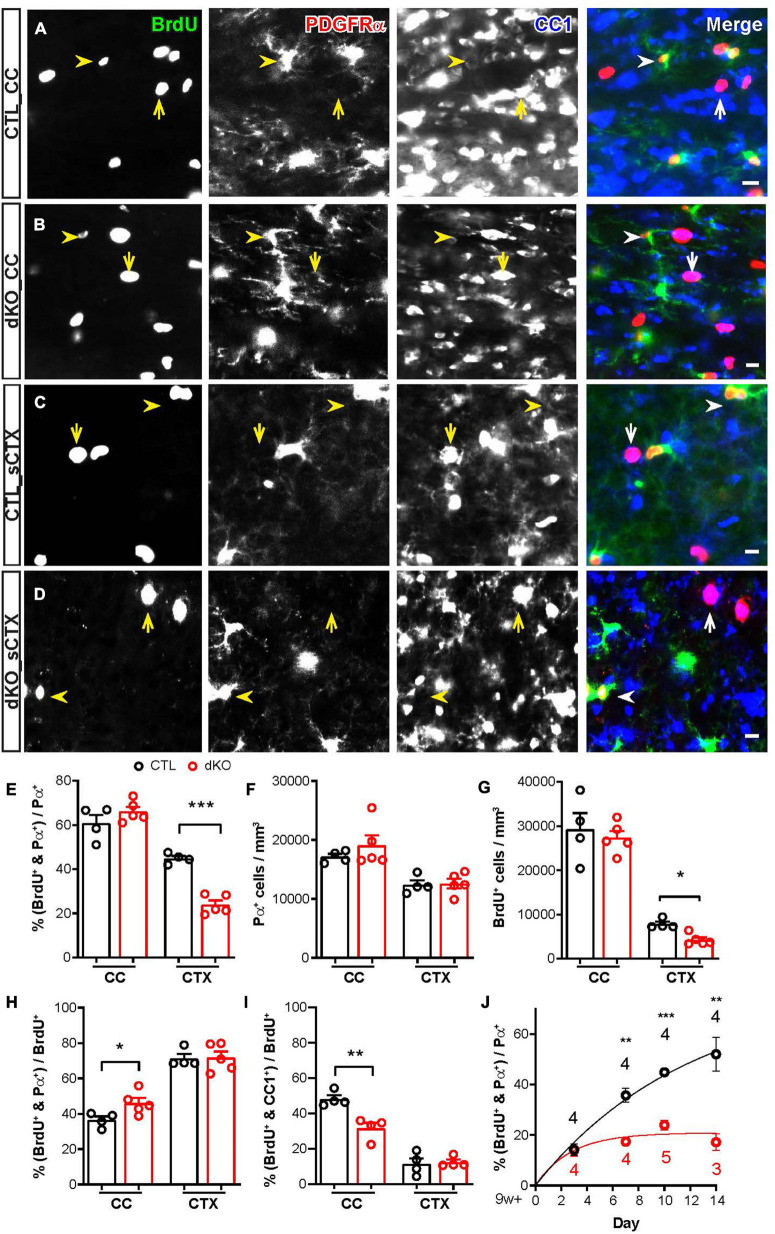
Conditional gene ablation of Cav1.2 and Cav1.3 inhibited the proliferation of NG2 glia in the cortex. **(A–D)** Epifluorescence images showing BrdU^+^ cells immunoactivity to PDGFRα or CC1 in the CC **(A,B)** and sCTX **(C,D)** of control and dKO mice. **(E)** The percentages of proliferating NG2 glia in the white and gray matter. In the gray matter, the number of proliferating NG2 glia from dKO mice was much lower than controls. Each datapoint represents results obtained from a single mouse. **(F)** Densities of the NG2 glia populations in control and mutant mice remained similar in various brain regions. **(G)** The density of proliferating NG2 glia in the CTX was significantly decreased. **(H,I)** The proportion of proliferating NG2 glia **(H)** in the CC was higher than controls, while the proportion of proliferated oligodendrocytes **(I)** in the CC was lower than controls. No difference could be detected in the gray matter. **(J)** The cumulative labeling of NG2 glia in the somatosensory cortex showed that a small proportion of still proliferating NG2 glia. The numbers in the graph represent mouse number. *p < 0.05; **p < 0.01; ***p < 0.001.

In the mature brain, uniformly distributed NG2 glia are the major proliferating cell population. Cumulative BrdU labeling of NG2 glia in the corpus callosum reached a plateau at ∼55% in 7–10 days, while in the cortex, the labeling increased linearly for 21 days until ∼40% of NG2 glia were BrdU^+^ (Rivers et al., 2008). To assess the self-renewal of Cav1.2 and Cav1.3 deficient NG2 glia, we performed an immunohistochemical analysis of mutant mice and their littermate controls treated with BrdU in drinking water for 10 days in the beginning of the 10th postnatal week. Platelet-derived growth factor receptor α (PDGFRα, short: Pα) and CC1 (a monoclonal antibody against adenomatous polyposis coli) expression were used to identify NG2 glia (Nishiyama et al., 1996; Huang et al., 2019) and mature oligodendrocytes (Zhu et al., 2012; Bin et al., 2016), respectively. We observed that some BrdU^+^ cells were Pα^+^ and CC1^–^ in the corpus callosum (CC, Figure 2A) and somatosensory cortex (sCTX, Figure 2C) of controls, indicating proliferating NG2 glia (arrowhead). Other BrdU^+^ cells were Pα^–^ and CC1^+^ (arrow) in all regions, indicating that these NG2 glia differentiated into oligodendrocytes after proliferation. In mutant mice, both Pα^+^CC1^–^BrdU^+^ and Pα^–^CC1^+^BrdU^+^ cells could be found in the CC (Figure 2B) and sCTX (Figure 2D). Our findings suggest that NG2 glia in both white and gray matter still keep dividing in the absence of L-type VGCCs.

However, the number of proliferating NG2 glia in the sCTX of dKO mice was much lower than in controls, while the number of proliferating NG2 glia in CC was unchanged (Figure 2E). The cell density of NG2 glia in all analyzed brain regions was not altered in dKO mice compared to their littermate controls (Figure 2F). In addition, we performed Caspase 3 staining to identify potentially increased cellular apoptosis. No difference was detected in the forebrain between dKO and control mice (data not shown), suggesting that deletion of Cav1.2 and Cav1.3 did not induce NG2 glial cell death in corpus callosum and cortex, respectively. Therefore, we performed a cumulative BrdU administration experiment in dKO mice and their littermate controls for 3, 7, and 14 days to assess the turnover of NG2 glia. The proportion of proliferating NG2 glia increased in controls (up to 53.0 ± 6.8% cells in 14 days post BrdU), whereas <20% of cortical NG2 glia in dKO mice were proliferating (only 17.8 ± 3.3% cells 14 days post BrdU, Figure 2J). The population of proliferating cells also did not increase in dKO mice, even though the BrdU treatment was prolonged. Altogether, our data revealed that Cav1.2 and Cav1.3 proteins are playing an important role in mediating proliferation of NG2 glia, but do not affect their survival.

Furthermore, by employing immunostaining against PDGFRα and CC1 to investigate the differentiation of proliferating NG2 glia in the absence of Cav1.2 and Cav1.3, we observed that the proportion of proliferating NG2 glia (Pα^+^CC1^–^BrdU^+^) in the corpus callosum of dKO mice was increased (Figure 2H), while the number of differentiated oligodendrocytes (Pα^–^CC1^+^BrdU^+^) was reduced remarkably (Figure 2I). This suggests that L-type VGCCs also have a function in regulating the oligodendrogenesis of proliferating subpopulation of NG2 glia especially in the white matter.

To evaluate whether loss of Cav1.2 and Cav1.3 in NG2 glia could impair their differentiation, we performed co-immunostainings against Pα and CC1 in hippocampal brain slices in 10 weeks old mice. The reporter recombination efficiency was about 50% in both white and gray matter (Supplementary Figure 1E). In the corpus callosum and cortex of controls, some GFP^+^ cells were Pα^+^ and CC1^–^ (arrowhead) and therefore identified as NG2 glia. The remaining GFP^+^ cells were Pα^–^ and CC1^+^ (arrow) and determined as mature oligodendrocytes (Supplementary Figures 1A,C), consistent with previous work (Huang et al., 2014). In dKO mice, both cell types, Pα^+^ CC1^–^ NG2 glia and Pα^–^ CC1^+^ oligodendrocytes were detected in corpus callosum and cortex (Supplementary Figures 1B,D). More than 80% of GFP^+^ cells in the corpus callosum expressed the oligodendrocyte specific marker CC1 in both control and dKO mice (CTL, 81.15 ± 1.72%, *n* = 6 mice; dKO, 82.14 ± 1.87%, *n* = 8 mice, Supplementary Figure 1F). In the cortex of dKO mice, 33.82 ± 1.43% of GFP^+^ cells were Pα^+^ NG2 glia and in control mice 27.36 ± 3.00%. 64.20 ± 1.55% of GFP^+^ cells in dKO mice were CC1^+^ oligodendrocytes, while 70.14 ± 2.55% of GFP^+^ cells in controls (Supplementary Figure 1G). Since no differences could be detected, these data indicate that the capability of NG2 glia to differentiate into oligodendrocytes was not influenced by deleting Cav1.2 and Cav1.3 proteins in white and gray matter.

### Loss of NG2 Glial Cav1.2 and Cav1.3 Changed Their Morphology

**FIGURE 3:**
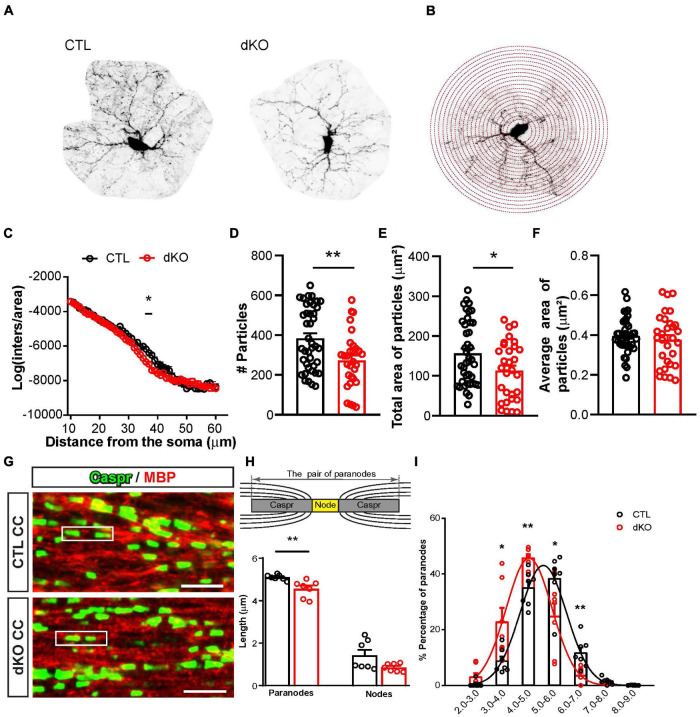
Conditional gene ablation of Cav1.2 and Cav1.3 resulted in a less complex morphology of NG2 glia and shortened paired paranodes. **(A)** A typical NG2 glia cell in layer II/III of cortex from CTL and dKO mice. **(B)** Sholl analysis with process intersections being counted every 1 μm. **(C)** In the range of 32–41 mm, i.e., far away from the soma, Cav1.2/1.3 deficient NG2 glia displayed less processes than controls. **(D–F)** The processes quantification using binary threshold analysis shows that Cav1.2/1.3 deficient NG2 glia had less particle-shaped varicosities **(D)**, which occupied a smaller total area **(E)** than control NG2 glia, while the average particle size was unaltered **(F)**. Each datapoint represents data from a single NG2 glial cell. **(G)** Co-immunolabeling of CASPR (green) and MBP (red) in the CC. Scale bar = 5 μm. **(H)** The paired paranodes in mutant mice were shorter than controls. Each datapoint represents data obtained from a single mouse. The upper depicts the quantification of paired paranodes. **(I)** The distribution of paired paranodes in dKO mice showed a left shift compared to controls. *p < 0.05; **p < 0.01.

To investigate the morphology of NG2 glia in the absence of Cav1.2 and Cav1.3, a Sholl analysis (Sholl, 1953) in combination with a binary particle analysis was performed on isolated Pα^+^GFP^+^ NG2 glia in layer II/III of the cerebral cortex. NG2 glia exhibit numerous and highly branched processes (Figure 3A). The Sholl analysis (Figure 3B) showed that Cav1.2/1.3 deficient NG2 glia had less intersections than controls in the distance between 32 and 41 μm away from the soma (Figure 3C), suggesting that Cav1.2/1.3 deficient NG2 glia lose some of their fine processes.

To confirm this finding, a binary particle analysis was applied. Particle size was defined as GFP-fluorescent areas of 1–20 pixel^2^ (0.05–4 μm^2^). Cav1.2/1.3 deficient NG2 glia had 41% less particles than controls (dKO, 272.7 ± 25.6 particles, *n* = 30 cells from 4 mice; CTL, 384.4 ± 24.4 particles, *n* = 41 cells from 3 mice. The total area of these particles from dKO mice was similarly reduced by 39% (dKO, 112.90 ± 13.31 μm^2^; CTL, 156.40 ± 12.52 μm^2^) (Figures 3D,E). No differences in the average size of these particles could be found (CTL, 0.40 ± 0.01 μm^2^; dKO, 0.38 ± 0.02 μm^2^) (Figure 3F). These data revealed that Cav1.2/1.3 deficient NG2 glia occupied less space in the cortex and their processes were shorter than controls with a less complex morphology. Altogether, it implies that mutant NG2 glia have fewer contacts with axons or other cell types, such as astrocytes.

The morphological changes suggested that also myelin structures could be affected as well, although Western blot analysis of myelin proteins (data not shown) and myelin basic protein (MBP) immunostainings (Supplementary Figures 2A,B) showed no differences as response to the removal of Cav1.2 and Cav1.3, neither in white nor in gray matter. The expression of contactin-associated protein (CASPR) is restricted to the paranodal regions of mature myelinated axons in the peripheral and central nervous system (Einheber et al., 1997). Therefore, we used CASPR and MBP co-staining to further investigate the myelinated structures in the absence of L-type VGCC (Figure 3G). We estimated the paired paranodal length by measuring CASPR staining (Figure 3H top). In the corpus callosum of dKO mice, the length of paired paranodes was significantly shortened as compared to controls, while no difference could be detected in the length of nodes of Ranvier (Figure 3H). The distribution of paired paranodes showed a shorter distribution (Figure 3I, shift to the left side). In addition, we examined paired paranodes in other white matter regions (spinal cord and optic nerve), but could not find differences there (Supplementary Figure 3 and Table 1). Our data suggest that the loss of Cav1.2 and Cav1.3 in NG2 glia affected the formation of callosal myelinated structures, while it had no impact in other CNS regions with compacted myelin (spinal cord and optic nerve).

**TABLE 1:** The length of paired paranodes in the white matter.

**Brain region**	**Paired paranodes (μm) CTL vs. dKO**	**Node of Ranvier (μm) CTL vs. dKO**	**# Mice CTL vs. dKO**
Corpus callosum	5.10 ± 0.05 vs. 4.56 ± 0.15	1.42 ± 0.26 vs. 0.85 ± 0.07	2,911 from 7 mice vs. 3,483 from 7 mice
Spinal cord	5.06 ± 0.14 vs. 4.94 ± 0.14	0.46 ± 0.07 vs. 0.36 ± 0.06	3,023 from 7 mice vs. 3,503 from 7 mice
Optic nerve	4.67 ± 0.10 vs. 4.58 ± 0.07	0.41 ± 0.02 vs. 0.38 ± 0.03	496 from 4 mice; dKO, *n* = 649 from 5 mice

To determine putative functional consequences of shortened paranodes, CAP of callosal axons were recorded in acute brain slices prepared from dKO mice and their littermate controls at 8–12 week-old mice. Previously, properties of callosal CAPs had been described as biphasic waves with an early component evoked by fast conducting myelinated axons (N1) and a later occurring component mainly from slower unmyelinated axons (N2) (Crawford et al., 2009). Callosal CAP recordings at five different distances between stimulus and recording electrodes from control and dKO mice were quantified and compared (Supplementary Figure 2D). Conduction velocity of myelinated callosal axons was calculated by linear regression with a value of 0.847 mm ± 0.046 ms^–1^ (*n* = 21 slices from 5 mice) in controls and 0.853 ± 0.042 ms^–1^ in dKO mice (*n* = 13 slices from 3 mice), whereas conduction velocity of unmyelinated axons was 0.311 ± 0.095 ms^–1^ in controls and 0.314 ± 0.093 ms^–1^ in dKO mice (Supplementary Figure 2D). No differences were detected in the conduction velocities of axons in the corpus callosum. The callosal CAP responses of both waves N1 and N2 increased proportionally upon the increment of stimulus currents in control and dKO mice (Supplementary Figure 2C). Obviously, the slight shortening of paranodal structures upon NG2-glial ablation of Cav1.2 and Cav1.3 is not sufficient to affect the conduction velocity of callosal axons.

### Ablation of Cav1.2 and Cav1.3 in NG2 Glia Affected Neuron-Glia Microcircuits

**FIGURE 4:**
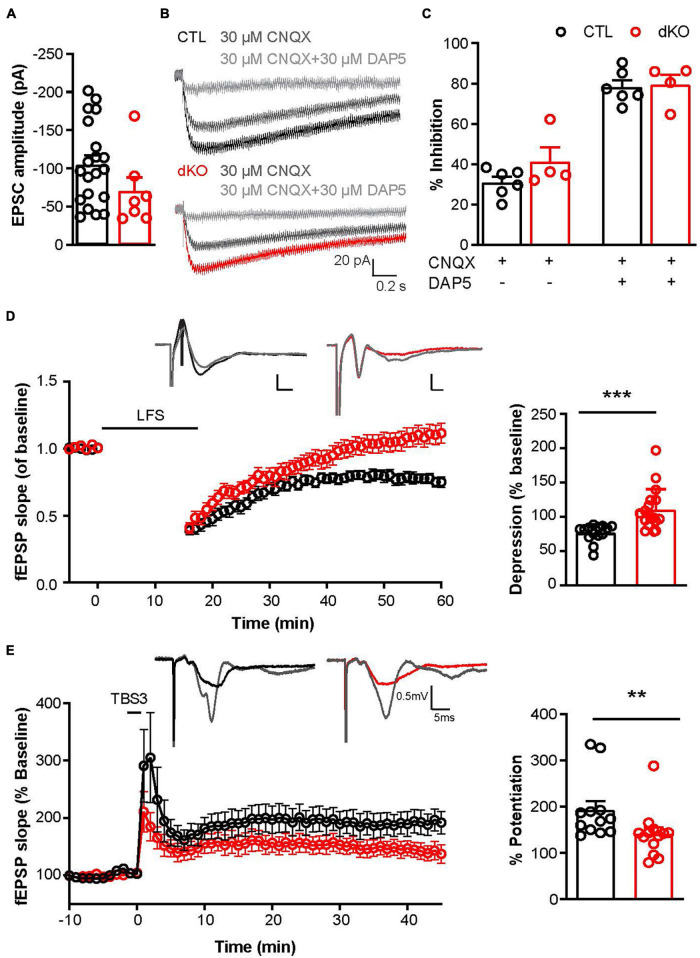
Functional consequences of the conditional L-type VGCCs gene ablation for NG2 glia-neuron interaction and synaptic plasticity. **(A)** Evoked excitatory postsynaptic currents (EPSCs) in cortical NG2 glia of CTL and dKO mice. **(B)** EPSC amplitudes of CTL and dKO mice. **(C)** Inhibition of EPSCs in CTL and dKO mice by antagonists of AMPARs and NMDARs. Each datapoint represents data obtained from a single NG2 glial cell. **(D)** Long-term depression (LTD) evoked by low-frequency stimulation (LFS) could be elicited in controls, but not in dKO mice. Each datapoint represents recordings from a single slice. More than three mice per group were analyzed. Scale bar, *x* = 2 ms, *y* = 0.5 mV. **(E)** Long-term potentiation (LTP) evoked by triple θ-burst stimulation (TBS3) in dKO mice were significantly decreased compared to controls. Scale bar, *x* = 5 ms, *y* = 0.5 mV. **p < 0.01; ***p < 0.001.

The formation of synapses is not restricted to neurons, also NG2 glia receive synaptic input (Bergles et al., 2010; Fröhlich et al., 2011; Sakry et al., 2011). Such neuron-NG2 glia synapses are down-regulated during the differentiation process into mature oligodendrocytes (Dimou and Gallo, 2015). NG2 glia also secrete cytokines or release transmitters to regulate neuronal networks (Birey et al., 2015). Therefore, we tested whether deletion of Cav1.2 and Cav1.3 also had an impact on synaptic input by recording EPSCs of NG2 glia in layer II/III of somatosensory cortex and stimulating presynaptic axons in layer V in the presence of the GABA_*A*_R (γ-Aminobutyric acid type A receptor) antagonist picrotoxin. Age-matched NG2-EYFP mice were used as controls. The EPSC amplitude of Cav1.2/1.3 deficient NG2 glia was −70.34 ± 17.94 pA (*n* = 7 cells from 3 mice). No differences were observed in controls (−105.30 ± 11.92 pA, *n* = 20 cells from 5 mice) (Figure 4A). Pharmacological inhibition using 6-cyano-7-nitroquinoxaline-2,3-dione (CNQX) and (2R)-2-amino-5-phosphonopentanoic acid (D-AP5), selective blockers for AMPAR and NMDAR currents, showed that EPSCs of cortical NG2 glia were mainly mediated by AMPARs and NMDARs (Figures 4B,C). No differences were observed between control and dKO mice indicating that the deletion of Cav1.2 and Cav1.3 did not alter the expression density of glutamate receptors on the membrane of NG2 glia.

It has been reported that a bidirectional crosstalk between NG2 glia and their adjacent cells such as neurons and astrocytes at neuron-NG2 glia synapses contributes to the regulation of neuronal networks. NG2 glia respond to neuronal signals by rising intracellular Ca^2+^, a potential signaling trigger by which NG2 glia may become an active player in neuron-glial circuits (Wigley et al., 2007). Based on our previous findings of decreased intracellular Ca^2+^ (Figure 1J) and less complex morphology (Figure 3) in mutant NG2 glia, we suspected that neuron-NG2 glia synapses might be disturbed and thereby could affect neuronal plasticity. To determine whether knockout of Cav1.2 and Cav1.3 from NG2 glia would influence the neuronal network, we performed field recordings of excitatory postsynaptic potentials (fEPSP) in the hippocampal CA1 by stimulating CA3 region. Acute brain slices were prepared from dKO mice and their littermate controls at 8–12 weeks. And indeed, long-term depression (LTD) evoked by low-frequency stimulation (LFS) could be elicited in controls, however not in dKO mice (CTL, 76.7 ± 3.4%, *n* = 14 slices from 6 mice; dKO, 110.2 ± 7.1%, *n* = 18 slices from 7 mice, Figure 4D). This LTD could be blocked by D-AP5 (data not shown). When 25 μM NMDA was applied in the bath for 7 min, LTD could be evoked in controls, but not in dKO mice (data not shown). These data indicate that the loss of Cav1.2 and Cav1.3 in NG2 glia impaired NMDA-dependent LTD in the hippocampus. Furthermore, by performing high-frequency stimulation in the hippocampal CA3, we could evoke long-term potentiation (LTP) in dKO mice, which was 27% lower than in controls (CTL, 192.6 ± 19.4%, *n* = 7 slices from 4 mice; dKO, 140.0 ± 14.2%, 11 slices from 5 mice, Figure 4E). All these data suggest that NG2 glia play an important role in neuronal plasticity regulated by the activation of NG2 glial L-type voltage-gated Ca^2+^ channels.

## Discussion

In oligodendrocyte lineage cells, L-type voltage-gated Ca^2+^ channels are highly expressed in progenitor cells and rapidly down-regulated when differentiating into mature oligodendrocytes, suggesting that L-type VGCCs mostly regulate the function of progenitor cells, namely NG2 glia. Influx of Ca^2+^ ions *via* the plasma membrane of NG2 glia induces Ca^2+^-induced Ca^2+^ release from the endoplasmic reticulum, followed by a rapid rise of intracellular Ca^2+^ and subsequently triggering a series of cellular activities at different time scales, ranging from immediate responses to neuronal signals to long-term processes such as differentiation, proliferation and migration. In this study, we used the tamoxifen-inducible CreERT2 system to investigate the functional roles of NG2 glia. Our results show that NG2 glia do not require the L-type VGCC subtypes Cav1.2 and Cav1.3 for their differentiation into mature oligodendrocytes or for myelination. In the gray matter cortex, removal of Cav1.2 and Cav1.3 led to a decrease of proliferating NG2 glia, but not in the white matter corpus callosum, thereby indicating a distinct heterogeneity of NG2 glia in different brain regions. In the absence of L-type VGCCs, NG2 glia lost parts of their complex morphology and alterations of paranodal myelin structures. Notably, inhibiting the intracellular Ca^2+^ rise by ablation of Cav1.2 and Cav1.3 selectively in NG2 glia consequently affected neuronal activities. Obviously, NG2 glial L-type VGCCs are parts of the modulatory system that determines the neuronal network activities.

### L-Type Voltage-Gated Ca^2+^ Channel Subtypes Cav1.2 and Cav1.3 Are Not Required for Differentiation Into Mature Oligodendrocytes and Subsequent Myelination

Our electrophysiological data showed that in the gray matter most NG2 glia expressed L-type VGCCs. The expression level of L-type VGCCs reached a peak in the first postnatal week, and then exhibited a decline in an age-dependent manner. In particular, Ca^2+^ currents decreased largely in young adult mice after weaning compared to early postnatal stages in the gray matter (Figure 1B). Therefore, we postulated that L-type VGCCs could play crucial roles in the behavior of NG2 glia, such as, differentiation and proliferation, specifically in the early postnatal stage. Hence, we administered tamoxifen to young mice at postnatal day 7 and 8 to remove Cav1.2 and Cav1.3 in NG2 glia at the peak expression level. However, our data showed that the differentiation of NG2 glia into mature oligodendrocytes was not affected, neither in white nor in gray matter. Similarly, the loss of Cav1.2 and 1.3 did not affect the expression of major myelin proteins such as MBP. However, the lengths of specific paranodal myelin structures were reduced in the absence of L-type VGCCs, though without affecting axon conduction velocity (Figure 3 and Supplementary Figure 2).

Our observations on the impact of L-type VGCCs on NG2 glial differentiation and subsequent myelination are not consistent with published data, where ablation of Cav1.2 using a BAC transgenic NG2-CreER mouse line resulted in reduced oligodendrocyte numbers at early postnatal periods. In corpus callosum, cortex and striatum, also myelin formation was strongly impaired 3 weeks after recombination (Cheli et al., 2016). We explain these diverging results by the use of different genetic mouse lines. Since we are using NG2-CreERT2 knock-in mice, which are less prone to transgenic artifacts, our observations appear to be more reliable than conclusions obtained from BAC transgenic mice with a less consistent recombination efficiency (Guo et al., 2021). In contrast, we still observed a rise of intracellular Ca^2+^ upon depolarization in Cav1.2/1.3 deficient NG2 glia, probably generated by a compensatory upregulation of T-type Ca^2+^ VGCCs after deletion of L-type Ca^2+^ VGCCs. In line with this observation, NG2 glial Ca^2+^ signals evoked by trains of 10 postsynaptic potentials (100 Hz) are mainly generated by low-voltage activated T-type Ca^2+^ channels (Sun et al., 2016). This might also explain the lack of strong myelin alterations in the dKO mice. Moreover, as we showed that the number of Cav-expressing NG2 glia in the corpus callosum was lower than in the cortical gray matter (Figure 1B). It can be considered that the other subpopulation of non-Cav expressing NG2 glia compensated the phenotype induced by Cav-expressing NG2 glia in the corpus callosum.

### Cav1.2 and Cav1.3 Are Playing Central Roles in Regulating the Proliferation of NG2 Glia

In the mature brain, NG2 glia are maintained mainly by local self-renewal, especially in the gray matter. Upon treatment of BrdU, we observed less proliferating NG2 glia in the Cav1.2/Cav1.3-dKO gray matter, while cell survival and apoptosis based on cleaved caspase 3-staining was not affected (Figure 2). Cumulative administration of BrdU indicated only 20% of cortical NG2 glia proliferation in dKO mice after a week compared to control cells. Obviously, L-type VGCCs strongly determine the proliferation NG2 glia in the gray matter.

However, using another BAC transgenic mouse line targeting OPCs (Pdgfrα-CreER), Cav1.2 deletion resulted in a loss of OPCs in the adult corpus callosum, a week after induction of recombination. OPC density could recover within 2 weeks by fast proliferation of surviving Cav1.2-deficient NG2 glia (Pitman et al., 2020). NG2 glia have been shown to become functionally heterogeneous in different brain regions and kinetically vary with age (Dimou and Gallo, 2015; Spitzer et al., 2019). Notably, the ablation of Cav1.2 in NG2 glia was performed in the adult brain (at postnatal 60). In our study, tamoxifen-dependent recombination was induced at a peak expression level of L-type VGCCs in the gray matter at postnatal days 7 and 8. In addition, our data demonstrated about 50% of NG2 glia expressing L-type Ca^2+^ channels in the corpus callosum with smaller Ca^2+^ currents at an earlier time point. We cannot exclude that callosal proliferation doesn’t require the modulation by L-type VGCC in the first 2 postnatal weeks. By virtue of shorter cell cycle of callosal NG2 glia (Young et al., 2013), their self-renewal occurs faster than that in the gray matter. Taken together, L-type VGCCs of NG2 glia mediate different aspects during the development of CNS maturation.

### Morphological Changes Induced the Impairment of Neuronal Networks?

NG2 glia play important roles in maintaining CNS homeostasis by mediating astroglial glutamate uptake and neuronal glutamatergic signaling (Bergles et al., 2010). NG2 glia can not only receive synaptic input from both glutamatergic and GABAergic neurons (Bergles et al., 2000; Lin and Bergles, 2004), but also secrete factors, regulate OPC development, and in turn might modulate the neuronal network (Birey et al., 2015; Sakry et al., 2015). NG2 glia lose synaptic input when they differentiate into oligodendrocytes with a decrease in glutamate receptor expression (Kukley et al., 2007, 2010; De Biase et al., 2010). Neuron-glia synapses undergo activity-dependent modifications to long-term potentiation (LTP) at excitatory synapses, which was induced at neuron-NG2 glia synapses involving Ca^2+^-permeable AMPA receptors on NG2 glia in the hippocampus (Ge et al., 2006). In addition, ablation of NG2 glia causes deficits in the excitatory glutamatergic synaptic transmission in the PFC of the adult brain (Birey et al., 2015). NG2 protein deletion results in a striking reduction in NMDA receptor dependent LTP in pyramidal neurons of the somatosensory cortex and diminished NMDA and AMPA receptor-dependent current amplitudes in these neurons (Sakry et al., 2014). This list of compelling evidence demonstrates the pivotal bidirectional crosstalk between NG2 glia and the surrounding neuronal network and underlines the novel physiological role of NG2 glia in regulating information processing at neuronal synapses.

Upon Cav1.2 and 1.3 deletion NG2 glia lost their complex morphology. It is tempting to speculate that this could affect neuron-NG2 glia synapses, and based on putative loss of contact NG2 glia would be isolated from the surrounding neuronal network. However, we observed that deletion of Cav1.2 and Cav1.3 channels did not alter EPSC amplitudes of NG2 glia and their expression density of glutamate receptors, which were still predominantly mediated by Ca^2+^ permeable AMPA receptors, consistent with previous studies (Ge et al., 2006). These results indicate that lack of L-type VGCCs does not interfere with formation and basic properties of neuron-glia synapses. Furthermore, neuronal communications require primarily intracellular Ca^2+^ rise *via* Ca^2+^ permeable AMPARs or voltage-gated Ca^2+^ channels as well as extracellular K^+^ depolarization. To investigate the L-type VGCC contributing to neuronal activities, we evaluated neuronal plasticity reflected by LTD and LTP in the hippocampus. Both hippocampal LTD and LTP were impaired after deletion of Cav1.2 and Cav1.3 in NG2 glia, indicating L-type VGCC as key regulators in modulating neuronal networks. However, the underlying mechanism still appears enigmatic. We hypothesize that NG2 glia could modulate neuronal plasticity by secreting specific growth factors or cytokines and making fast feedback to neurons, initiated by intracellular Ca^2+^ rise though the activation of L-type VGCCs.

In summary, this work utilizes the simultaneous removal of L-type VGCC subtypes Cav1.2 and Cav1.3 to investigate the functional roles of NG2 glia. We could show the contribution of Cav1.2 and Cav1.3 in regulating the proliferation of NG2 glia. Ca^2+^ influx into NG2 glia through the activation of L-type VGCCs could consequently contribute to modulate neuronal activities.

## Data Availability Statement

The raw data supporting the conclusions of this article will be made available by the authors, without undue reservation.

## Ethics Statement

The animal study was reviewed and approved by the “Landesamt für Gesundheit und Verbraucherschutz” in Saarbrücken/Germany [animal license numbers: 72/2010, 65/2013, 34/2016 and FKI_cervical dislocation (§ 4)].

## Author Contributions

NZ and FK developed this project and wrote the manuscript. NZ carried out most of the experiments (electrophysiology, immunohistochemistry, and confocal imaging) and data analysis as well as figure preparation. WH generated the NG2-CreERT2 mice and contributed to the experimental design and the Western blot analysis. BC performed the morphological analysis of NG2 glia. FK and AS provided the supervision. All authors contributed to this article and approved the submitted version.

## Conflict of Interest

The authors declare that the research was conducted in the absence of any commercial or financial relationships that could be construed as a potential conflict of interest.

## Publisher’s Note

All claims expressed in this article are solely those of the authors and do not necessarily represent those of their affiliated organizations, or those of the publisher, the editors and the reviewers. Any product that may be evaluated in this article, or claim that may be made by its manufacturer, is not guaranteed or endorsed by the publisher.

## Supplementary Material

Supplementary Figure 1 NG2 glia still differentiate into oligodendrocytes in mutant mice after conditional Cav1.2/Cav1.3-gene ablation. **(A–D)** Mutant cells exhibited immunoreactivity to PDGFRα or CC1 in the corpus callosum (CC) and somatosensory cortex (sCTX) of CTL and dKO mice after tamoxifen administration. Scale bars = 10 μm. **(E)** The recombination efficiency of CAG-EGFP reporter mice. **(F,G)** The percentages of Pα^+^ GFP^+^ and CC1^+^ GFP^+^ cells were in the CC and CTX between controls and dKO mice.

Supplementary Figure 2 **(A)** Epifluorescent images of immunostainings against MBP (red) and nuclear DAPI (blue) staining in controls and mutant mice. Scale bar = 500 μm. **(B)** Quantification of MBP fluorescent intensities in different brain regions of mutant mice and controls during development. **(C,D)** Stimulus responses **(C)** of myelinated axons (N1) and non-myelinated axons (N2) and axon conduction velocity **(D)** estimated by compound action potential (CAP) recordings in the CC.

Supplementary Figure 3 Ablation of Cav1.2 and Cav1.3 did not affect myelin structures in spinal cord and optic nerve. **(A–D)** Confocal images showing immunostaining against Caspr and MBP in the spinal cord (SP, **A,B**) and optic nerve (OP, **C,D**) of control and dKO mice. **(E)** Lengths of paired paranodes were quantified in the spinal cord. Each datapoint represents one mouse. **(F)** Lengths of paired paranodes determined in the optic nerve.
