# Supplementary figures and images for: L-Type Ca2+ Channels of NG2 Glia Determine Proliferation and NMDA Receptor-Dependent Plasticity
Source: Front Cell Dev Biol. 2021 Oct 21;9:759477. doi: 10.3389/fcell.2021.759477 (PMC8567174; doi:10.3389/fcell.2021.759477)

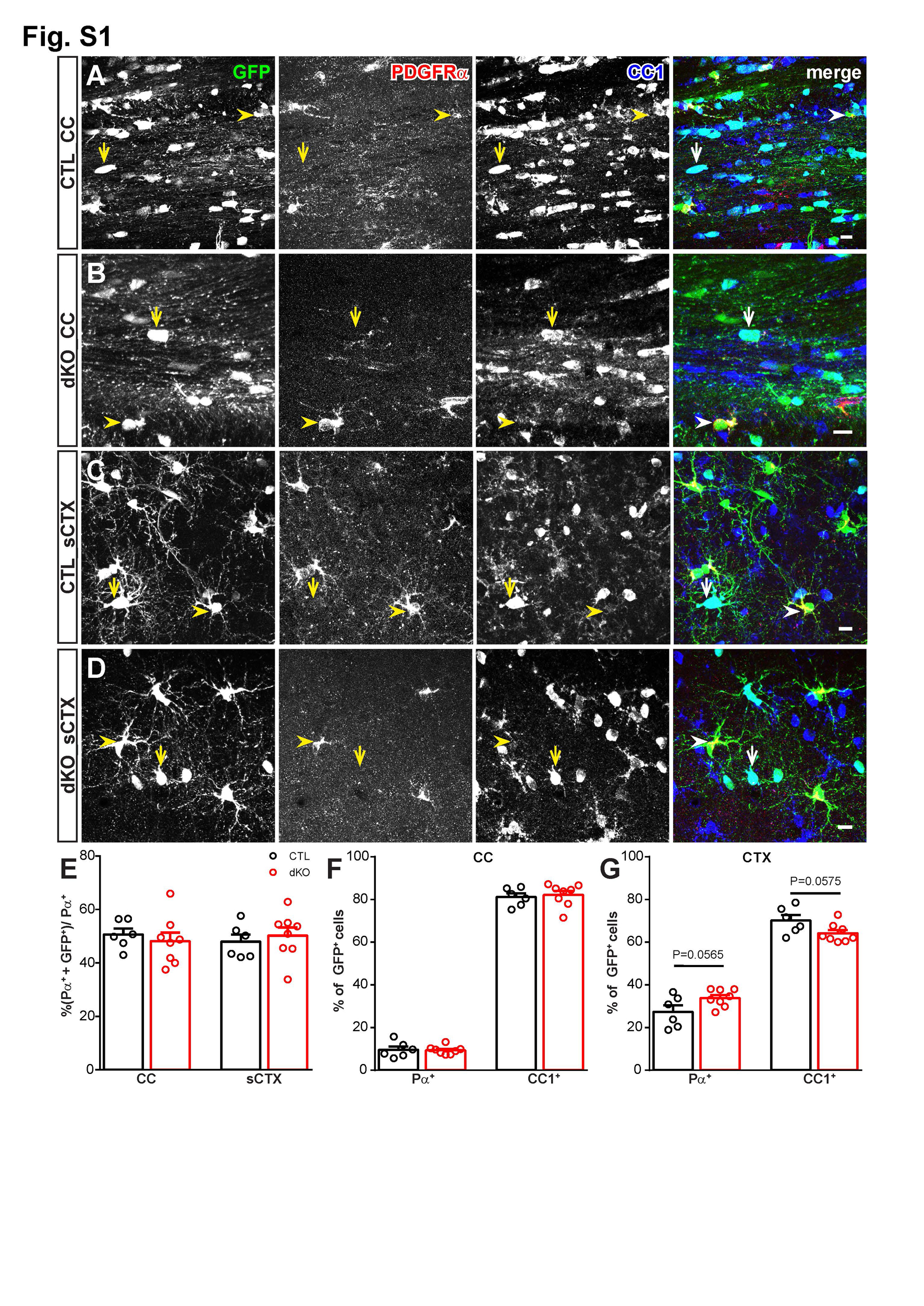

Supplement: Supplementary Figure 1 — NG2 glia still differentiate into oligodendrocytes in mutant mice after conditional Cav1.2/Cav1.3-gene ablation. (A–D) Mutant cells exhibited immunoreactivity to PDGFRα or CC1 in the corpus callosum (CC) and somatosensory cortex (sCTX) of CTL and dKO mice after tamoxifen administration. Scale bars = 10 μm. (E) The recombination efficiency of CAG-EGFP reporter mice. (F,G) The percentages of Pα+ GFP+ and CC1+ GFP+ cells were in the CC and CTX between controls and dKO mice. [file Image_1.jpeg]

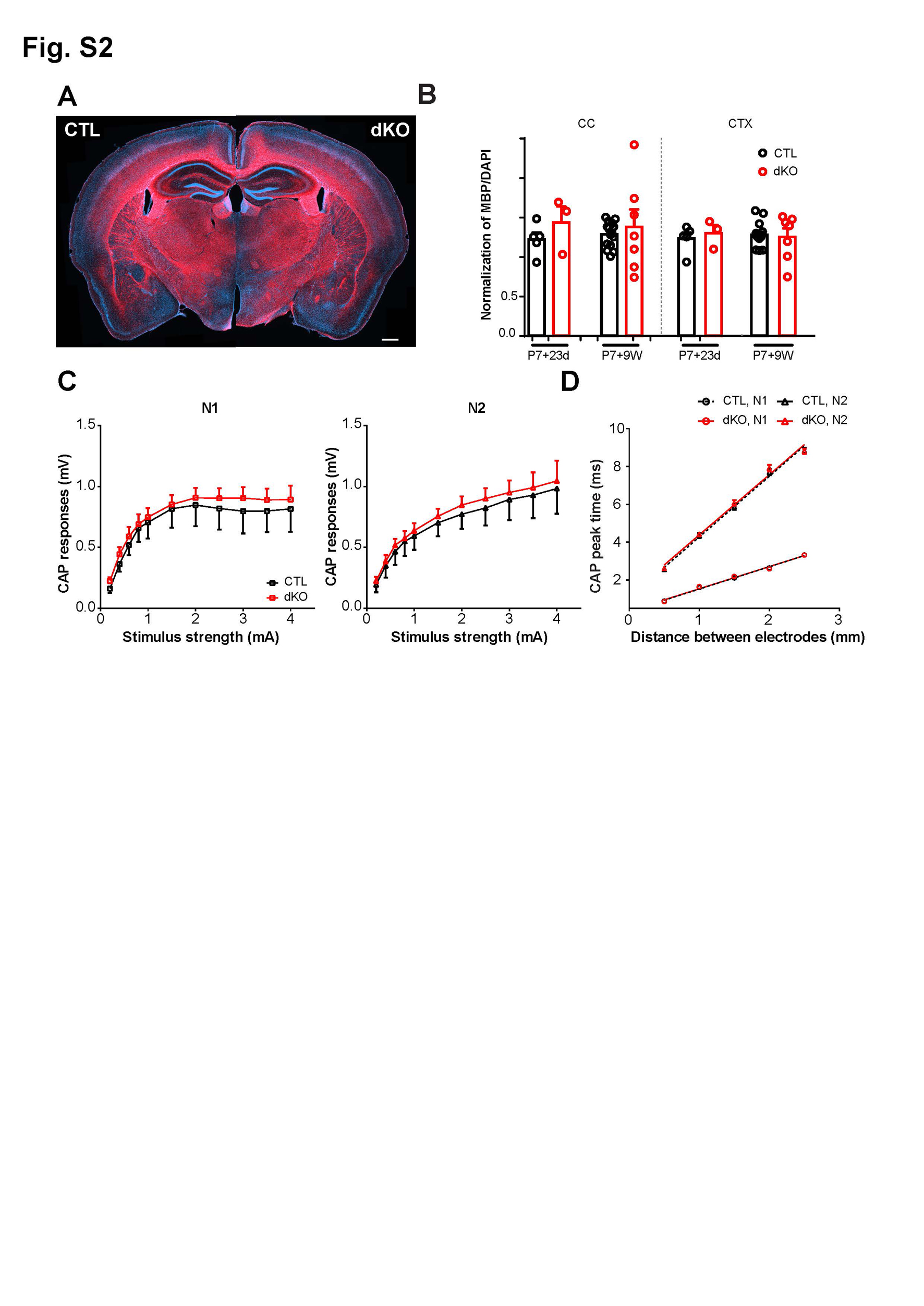

Supplement: Supplementary Figure 2 — (A) Epifluorescent images of immunostainings against MBP (red) and nuclear DAPI (blue) staining in controls and mutant mice. Scale bar = 500 μm. (B) Quantification of MBP fluorescent intensities in different brain regions of mutant mice and controls during development. (C,D) Stimulus responses (C) of myelinated axons (N1) and non-myelinated axons (N2) and axon conduction velocity (D) estimated by compound action potential (CAP) recordings in the CC. [file Image_2.jpeg]

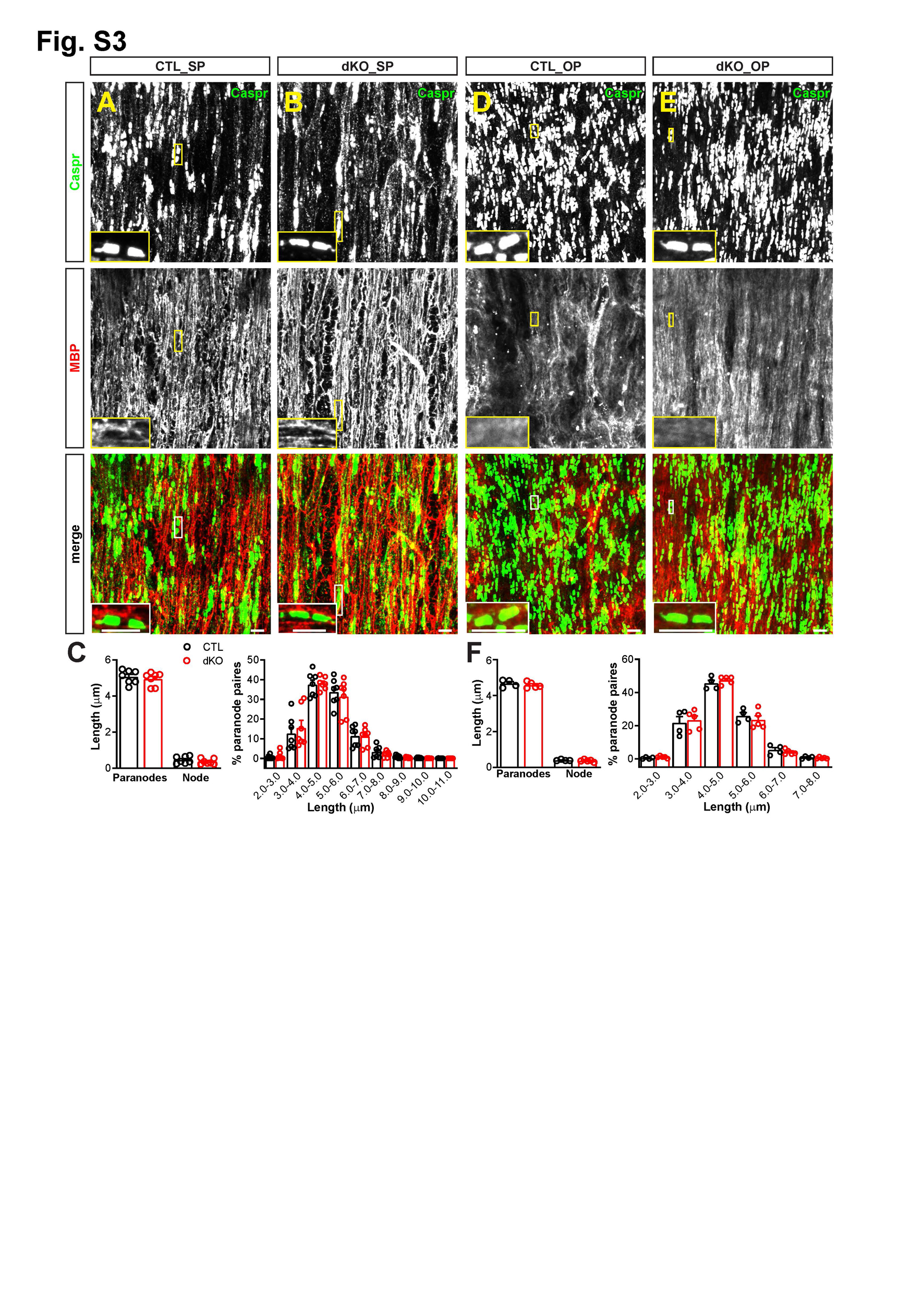

Supplement: Supplementary Figure 3 — Ablation of Cav1.2 and Cav1.3 did not affect myelin structures in spinal cord and optic nerve. (A–D) Confocal images showing immunostaining against Caspr and MBP in the spinal cord (SP, A,B) and optic nerve (OP, C,D) of control and dKO mice. (E) Lengths of paired paranodes were quantified in the spinal cord. Each datapoint represents one mouse. (F) Lengths of paired paranodes determined in the optic nerve. [file Image_3.jpeg]
